# Editorial: Recent advances in measuring and controlling synaptic communication

**DOI:** 10.3389/fncel.2023.1289874

**Published:** 2023-09-21

**Authors:** Jacopo Lamanna, Mattia Ferro, Emanuele Cocucci

**Affiliations:** ^1^Center for Behavioral Neuroscience and Communication (BNC), Vita-Salute San Raffaele University, Milan, Italy; ^2^Faculty of Psychology, Vita-Salute San Raffaele University, Milan, Italy; ^3^Department of Psychology, Sigmund Freud University, Milan, Italy; ^4^Division of Pharmaceutics and Pharmacology, College of Pharmacy, The Ohio State University, Columbus, OH, United States

**Keywords:** synaptic transmission, synaptic plasticity, exo-endocytosis, neurotransmission, activity labeling, optogenetics, ultrastructural analysis, quantal analysis

## Introduction

Synapses represent a unique neuronal compartment specialized in communication. The morpho-functional investigation of the synapse has always been hindered by its tiny size and high density of molecular elements, but specific technological and methodological advances helped us to reduce these limitations. As the matter of fact, in recent years, we have witnessed the continuous development of new methods allowing measuring and controlling synaptic activation both *in vitro* and *in vivo* (Lamanna et al., 2022). These include new genetically encoded sensors of synaptic exo-endocytosis (Ferro et al., 2017; Liu et al., 2021) and neurotransmitter release (Helassa et al., 2018; Patriarchi et al., 2018), but also engineered synaptic proteins able to control synaptic transmission (Won et al., 2021). Furthermore, new promising tools allow changing the functional properties of synapses in a plasticity-like manner (Goto et al., 2021).

All these methodological advances are likely to generate unprecedented knowledge about the dynamics of synaptic transmission and plasticity at several levels of the nervous system. Nevertheless, in most cases, the implementation of these new methods remains technically demanding, likely due to the high complexity of their operating principle (Glasgow et al., 2019; Lamanna et al., 2022). Hence, it would be worth refining and potentiating these tools to extend the range of experimental settings for their application. In addition, more classical and established approaches, such as electrophysiology, computational modeling and ultrastructural imaging, can be further implemented, e.g., by using alternative tools (Zhang et al., 2023) or advanced analysis approaches (Soares et al., 2019), to gather deeper insights into the physiology of neurotransmission.

In this Research Topic, we collected studies that validate, refine, or apply in an effective way advanced tools and approaches with the aim to investigate synaptic communication.

## Development and refinement of novel sensors and actuators

To extend our capabilities of sensing and/or controlling synaptic activity, it is worth to improve more effective probes, either of chemical origin or genetically encoded, but also to improve the experimental design and experimental setting to obtain more complete data and finally implement exhaustive pipelines for data analysis which often requires the development of dedicated software.

In this Research Topic, Seidenthal et al. describe the development and validation of the pOpsicle system, which combines recently developed variants of both pH-sensitive fluorescent proteins and channelrhodopsin, in order to simultaneously stimulate neuronal activity and measure exocytosis in the *Caenorhabditis elegans* using an all-optical approach.

Furthermore, Christensen et al. characterize a recently developed fluorescent reporter of neuropeptide Y (NPY) based on G-protein activation, evaluating its suitability to detect endogenous NPY release in primary neuronal cultures, as well as its technical limitations.

Utsumi et al. exploited voltage-sensitive dyes to characterize the electrophysiological response of seizure-inducing agents on *in vitro* hippocampal preparations; using this approach, the authors distinguish layer-specific activations as postsynaptic depolarizations or action potential activity, thus providing an effective tool for pharmacological testing of new compounds.

Finally, Rindner and Lur address the problem of cross-talk activation of opsins with scarcely-separated stimulation spectra in the same specimen and provide a method for estimating the acceptable range of stimulus intensity in multicolor optogenetics.

## Extending the toolkit for the electrophysiological analysis of the synapse

Established electrophysiological techniques are routinely applied to either synapses, single neurons, or brain regions to investigate neuronal communication and brain connectivity. Nevertheless, addressing complex scientific questions or investigating specific circuits may require more advanced and tailored analytical approaches. In addition, computational modeling and *in silico* simulations are increasingly necessary to design new experiments and generalize the findings.

Krotov et al. exploited a recently developed electrophysiological approach based on anodal block of myelinated Aβ/δ-fibers to selectively activate C-afferents of the rodent spinal cord, aimed to investigate presynaptic inhibition driven by both types of afferents: they show differential characteristics in terms of segments involved and reciprocity of this phenomenon.

Yoo et al. developed a recurrent synaptic network computational model to assess the effects of the different resting membrane potential (RMP) levels they found in neurons from *in vitro* preparations of prefrontal and posterior parietal cortices. The authors provide evidence for a role of RMP in defining the differential activity regimes of these regions, with implications for cognitive functioning, and suggest a role of NMDA receptors in generating RMP differences.

Lumeij et al. extend the applicability of a combined variance analysis approach based on inverse square of coefficient-of-variation and variance-to-mean ratio of synaptic transmission. Based on both simulations and experimental characterization of β-amyloid-induced synaptic depression, they show that this approach is effective for identifying the locus of change of synaptic strength even when quantal parameters are not assumed as uniform.

## Advances in ultrastructural imaging of the synapse

Due to the very limited size of the synaptic bouton, electron microscopy (EM) represents the best tool to morphologically characterize this compartment. Nevertheless, the simultaneous localization of multiple molecular and structural targets is technically challenging.

To address this issue, Kang et al. implemented a protocol for triple labeling of inhibitory and excitatory synapses in the pre-Bötzinger complex. They exploited, on the one hand, peroxidase and immunogold immunocytochemistry for targeting somatostatin and neurokinin 1, respectively, and, on the other, cytochrome oxidase histochemistry to characterize mitochondrial dynamics. Their investigation provides interesting insights about ultrastructural dynamics relevant to respiratory physiology.

Given the increasing number of approaches that exploit EM to study the synaptic function, Maiellano et al. discuss recent studies that exploited volume electronic microscopy, that can provide precise tomographic reconstruction of the synaptic bouton, and characterize the relationship between the geometry of postsynaptic compartments of central excitatory synapses and their functional state, in relation to synaptic plasticity phenomena.

## Concluding remarks

In conclusion, this Research Topic is a collection of multifarious studies, where very different methodological approaches are developed, refined, or effectively applied to investigate neuronal communication and synaptic physiology. Importantly, these works show how both established and recently developed techniques can be further implemented and tailored to answer important questions in the space of neurophysiology. We believe that this Research Topic will contribute to future neuroscientific research by providing novel approaches and useful insights to generate new ideas.

## Author contributions

JL: Writing—original draft, Writing—review and editing. MF: Writing—original draft, Writing—review and editing. EC: Writing—review and editing.
